# Effect of rutin on oxidative DNA damage in PC12 neurons cultured in nutrients deprivation condition

**DOI:** 10.22038/IJBMS.2020.31832.7657

**Published:** 2020-03

**Authors:** Marjan Nassiri-Asl, Ahmad Ghorbani, Sahar Salehisar, Elham Asadpour, Hamid Reza Sadeghnia

**Affiliations:** 1 Department of Pharmacology, School of Medicine, Shahid Beheshti University of Medical Sciences, Tehran, Iran; 2 Pharmacological Research Center of Medicinal Plants, Mashhad University of Medical Sciences, Mashhad, Iran; 3 Department of Pharmacology, Faculty of Medicine, Mashhad University of Medical Sciences, Mashhad, Iran; 4 Anesthesiology and Critical Care Research Center, Shiraz University of Medical Sciences, Shiraz, Iran; 5 Division of Neurocognitive Sciences, Psychiatry and Behavioral Sciences Research Center, Mashhad, University of Medical Sciences, Mashhad, Iran

**Keywords:** Apoptosis, DNA, Oxidative stress, PC12, Rutin

## Abstract

**Objective(s):**

Rutin is a flavonoid with potent antioxidant property, which exhibited cytoprotective effects in several models of neuronal injury. This work aimed to examine whether rutin can protect neurons against oxidative DNA damage caused by serum/glucose deprivation (SGD) as an in vitro model of neurodegeneration and ischemia.

**Materials and Methods:**

The PC12 cells were cultured for 2 hr in normal culture medium containing different concentrations of rutin or α-tocopherol (positive control) and then further incubated for 12 hr in SGD condition. Then, cell viability, DNA fragmentation, lipid peroxidation, generation of reactive oxygen species (ROS), and the expression of proteins involved in apoptosis were determined.

**Results:**

The SGD condition significantly decreased viability of the cells, which was accompanied by a significant rise in the generation of ROS and lipid peroxidation. Rutin enhanced the viability of PC12 cells in SGD condition and reduced the production of ROS and lipid peroxidation. In addition, rutin decreased DNA damage and inhibited apoptotic cell death by decreasing the levels of proapoptotic proteins (Bax, caspase-3, caspase-9) and increasing the level of anti-apoptotic protein Bcl-2.

**Conclusion:**

This study demonstrated that rutin inhibits oxidative DNA damage and neuronal death induced by nutrients deprivation condition. Further studies may warrant the use of rutin as an appropriate neuroprotective agent for ischemic attacks and other neurodegenerative disorders.

## Introduction

Neuronal damage following stroke or transient ischemic attacks results from multiple injury processes including excitotoxicity, ionic imbalances, acidotoxicity, oxidative stress, and inflammation (1, 2). Neurons are obviously vulnerable to oxidative stress-induced injury because of high energy demand and having low capacity of endogenous antioxidants (1, 3). In addition, increased level of reactive oxygen species (ROS) and subsequent oxidative damage of DNA are considered to be at the core of mechanisms leading to neurodegeneration following ischemia (2, 4). Therefore, use of cytoprotective phytochemicals with potent antioxidant activity might be a promising approach for enhancing neuronal survival in ischemic attacks and other neurodegenerative disorders.

Rutin, a flavonoid phytochemical found in several plant species, is one of the most potent antioxidant, which exhibited cytoprotective effect against oxidative stress (5, 6), inflammation (7), and chemical-induced toxicity (8). Recent experimental studies have revealed its neuroprotective effect in several models of neuronal injury including seizure, Alzheimer’s disease, diabetic neuropathy, and spinal cord damage (9-12). In an experimental model of subarachnoid hemorrhage, rutin inhibited neuro-inflammation, the blood-brain barrier damage, and neurological deficits (13). Rutin also reduced the infarct size and improved spatial memory, and behavioral deficits in cerebral ischemia models (14, 15).

The present work aimed to examine whether rutin can protect PC12 neuronal cells against DNA damage and apoptosis induced by serum/glucose deprivation (SGD). Since the fundamental pathophysiology of the ischemic attacks is the reduction of supply of oxygen, glucose, and the growth factors toward neurons, the SGD is generally considered as an *in vitro* model for examining novel neuroprotective agents and for the elucidation of the underlying molecular mechanisms (16-18). The PC12 cell line, that are of rat pheochromocytoma origin, is a widely accepted model for investigations pertaining to the function and survival of neurons in different pathological states (17, 19).

## Materials and Methods

***Materials***

Fetal bovine serum, glucose-free Dulbecco’s Modified Eagles Medium (DMEM), and DMEM containing high glucose were bought from Gibco (Carlsbad, CA, USA). Rutin was purchased from Fluka (St. Gallen, Switzerland). The PC12 cell line was bought from Pasteur Institute (Tehran, Iran). Normal melting point (NMP) and low melting point (LMP) agaroses were purchased from Fermentas (Glen Burnie, MD, USA). Tris (hydroxymethyl) aminomethane (Trizma base), ethylene diaminetetraacetic acid disodium salt (Na_2_EDTA), and sodium lauroyl sarcosinate (sarkosyl) were bought from Merck (Darmstadt, Germany). Antibodies against Bax, Bcl-2, caspase-3, and caspase-9 were bought from Cell Signaling Technology (Danvers, MA, USA). Protease inhibitor cocktail, methylthiazolyldiphenyl tetrazolium (MTT), bicinchoninic acid protein assay kit, and dichlorodihydrofluorescein diacetate (H_2_DCF-DA) were obtained from Sigma (St Louis, MO, USA).

***Cell culture and treatment***

The PC12 cells were cultured in 96-well culture plates (5×10^3^ cells per well) and maintained overnight in normal medium (high-glucose DMEM supplemented with 10 % fetal bovine serum). To determine non-toxic concentrations of rutin, the cells were incubated with 0-800 µM rutin in the normal medium for 24 hr. In a separate experiment, to assess the neuroprotective activity of rutin, the cells were first pre-incubated for 2 hr with 0-200 µM of rutin or 100 µM α-tocopherol (positive control), and then the normal medium was replaced by DMEM free of glucose and serum. Then, the cells were maintained in this SGD condition for 12 hr (20).

***Cell viability assay***

The cell viability was determined by MTT colorimetric test. After incubation in SGD condition, the MTT dye was added to the medium at the final concentration of 0.05%. After 4 hr, the precipitate of formazan was dissolved in dimethyl sulfoxide and the absorbance was read at 570 nm (21).

***Measurement of ROS***

The level of intracellular ROS was evaluated using H_2_DCF-DA, as a fluorescent probe. H_2_DCF-DA diffuses through the cell membrane and is hydrolyzed to H_2_DCF by intracellular esterases. Then, H_2_DCF is rapidly oxidized to dichlorofluorescein in the presence of ROS. Briefly, the PC12 cells were cultured in 96-well plates (1×10^4^ cells per well) and then incubated for 30 min with 10 µM of H_2_DCF-DA at 37 ^°^C. After washing with warm phosphate-buffered saline, the cells were pre-incubated for 2 hr with rutin, and then further incubated for 12 hr in SGD condition. The fluorescence intensity of dichlorofluorescein was determined at excitation/emission wavelength of 485/530 nm using a fluorescence plate reader (Perkin Elmer 2030, Multilabel reader, Finland).

***Lipid peroxidation assay***

The PC12 cells were cultured in 12-well plates (1×10^5^ cells per well), pretreated for 2 hr with rutin, and incubated for 12 hr in SGD condition. The lipid peroxidation was assessed by determining the level of malondialdehyde (MDA), the final product of lipid peroxidation. After incubation in SGD condition, the cells were scraped into a tube containing 1 ml trichloroacetic acid (2.5%) and centrifuged for 2 min at 13000 g. The supernatant (500 µl) was mixed with 400 µl of trichloroacetic acid (15 %) and 800 µl of thiobarbituric acid 0.67 % and butylated hydroxytoluene 0.01 %. Then, the mixture was vortexed, boiled for 20 min, and centrifuged for 10 min at 2500 rpm. The fluorescence intensity of supernatant was measured at an excitation/emission of 530/550 nm. The content of protein in samples was determined by bicinchoninic acid kit, and the MDA level was normalized to the level of protein.

***Comet assay (alkaline single cell gel electrophoresis)***

The PC12 cells were cultured in 6-well plates (5×10^5^ cells per well), pretreated with rutin for 2 hr, and incubated for 12 hr in SGD condition. Then, the cells were harvested and layered over a microscope slides and incubated overnight in cold lysis solution (10 mM Trizma, 100 mM Na_2_EDTA, 2.5 mM NaCl, 10% dimethyl sulfoxide, 1% sarkosyl, 1% Triton X-100, pH=10). The slides were placed for 40 min on a gel electrophoresis platform containing an alkaline solution (1 mM Na_2_EDTA, 300 mM NaOH, pH>13) and subjected to electrophoresis (25 V, 300 mA, 30 min). Then, the slides were washed with Trizma solution (pH=7.5) and incubated with ethidium bromide. Normal cells had intact nucleus without a tail, while damaged cells showed an appearance of tail (22). The percent of DNA appeared in the comet form (% tailed DNA), was determined using CASP image analysis software.

***Western blotting analysis***

The cells were cultured in T-25 flasks (1×10^6^ cells/well), pretreated with rutin for 2 hr, and incubated for 12 hr in SGD condition. Then, the cells were incubated with lysis buffer containing 150 mM NaCl, 50 mM Tris-HCl, 2 mM EDTA, 1 mM sodium orthovanadate, 5 mM sodium fluoride, 1% Nonidet P-40, and protease inhibitor. The cell lysates were centrifuged for 20 min at 13000 g, and the protein level of supernatants was measured by bicinchoninic acid protein assay kit. Equal amounts of protein from each treatment group were mixed with loading buffer and then boiled for 5 min. After electrophoresis, the separated proteins were moved to polyvinylidene fluoride membrane. Then, the membrane was placed in blocking buffer (150 mM NaCl, 50 mM Tris/HCl, 5% skimmed milk, and 0.1% Tween 20) and the blots were probed with the antibodies for overnight. The bound antibody was detected using a secondary antibody (horseradish peroxidase-conjugated goat anti-rabbit) and an enhanced chemiluminescence system. Gel-Pro Analyzer Software (Media Cybernetics) was used for analyzing density of the bands.

***Statistical analysis***

Results were compared using one-way analysis of variance. Tukey’s *post hoc* test was performed to reveal the specific pairs of treatment groups by which significant differences occurred. The results are presented as the mean±SEM. The *P*-values less than 0.05 were considered to be statistically significant. 

## Results

***Effect of rutin on cell viability***

**Figure 1:**
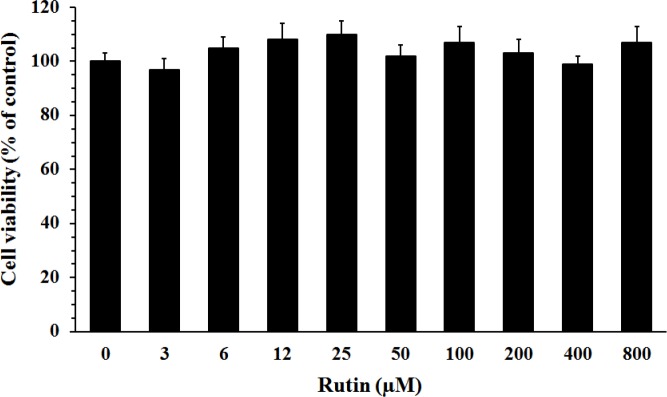
Effect of rutin on the viability of PC12 cells. The cells were treated for 24 hr with rutin in normal culture medium, and the percent of cell viability was normalized against the control (rutin 0 µM). Rutin had no significant effect on the cell viability even at high concentrations. Data are mean±SEM (n = 6)

To determine non-toxic concentrations of rutin, PC12 cells were incubated with increasing range of rutin concentrations (3-800 µM) for 24 hr in normal culture media. The results showed that rutin had no significant cytotoxicity even at high concentrations (Figure 1).

***Effect of rutin on cell death induced by SGD***

**Figure 2:**
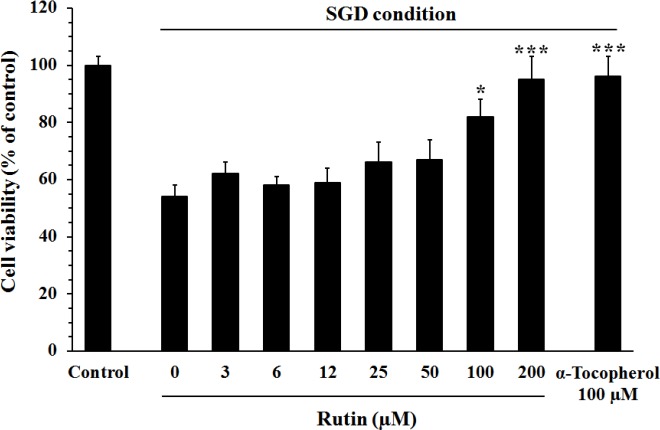
Effect of rutin on the viability of PC12 cells in serum/glucose deprivation (SGD) condition. The cells were pretreated with rutin for 2 hr and then exposed for 12 hr to SGD condition. The percent of cell viability was normalized against control cells cultured in the normal culture medium. Pretreatment with rutin significantly attenuated the SGD-induced cell death. Data are mean±SEM (n=6). *P<0.05, ***P<0.001 as compared to untreated cells (rutin 0 µM) cultured in SGD condition

The culture of PC12 cells in SGD condition significantly decreased cell viability, compared to the cells cultured in normal medium (54±4 % *vs* 100±3 %, *P*<0.001). Pretreatment with α-tocopherol or rutin significantly attenuated the SGD-induced cell death (Figure 2). The percent of viability in the cells pretreated with 100 µM and 200 µM of rutin was 82±6% (*P*<0.05, *vs* vehicle), and 95±8% (*P*<0.001), respectively.

***Effect of rutin on ROS generation***

**Figure 3:**
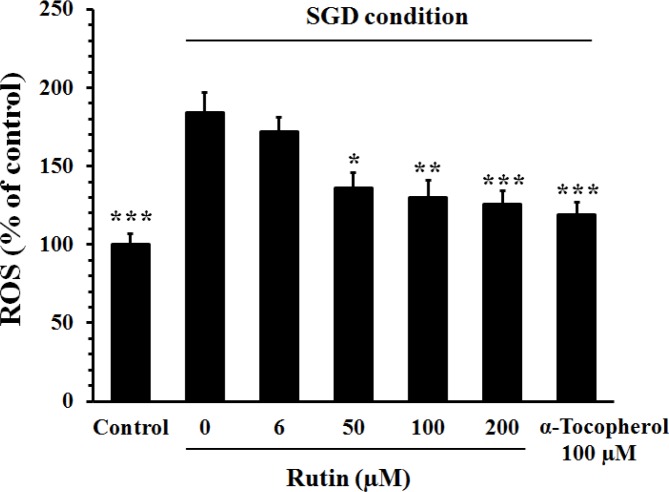
Effect of rutin on the intracellular reactive oxygen species (ROS) content in PC12 cells cultured in serum/glucose deprivation (SGD) condition. The cells were pretreated with rutin for 2 hr and then exposed for 12 hr to SGD condition. The percent of ROS content was normalized against control cells cultured in the normal culture medium. Pretreatment with rutin significantly decreased the SGD-induced ROS accumulation in PC12 cells. Data are mean±SEM (n=6) *P<0.05, **P<0.01, and ***P<0.001 as compared to untreated cells (rutin 0 µM) cultured in SGD condition

The level of intracellular ROS in the cells cultured in SGD condition was significantly higher than those cultured in normal condition (184±13% *vs* 100±7%, *P*<0.001). Pretreatment with rutin decreased the SGD-induced ROS accumulation in a concentration-dependent manner (Figure 3). This effect of rutin at 200 µM was comparable with that of α-tocopherol (100 µM). 

***Effect of rutin on lipid peroxidation***

**Figure 4:**
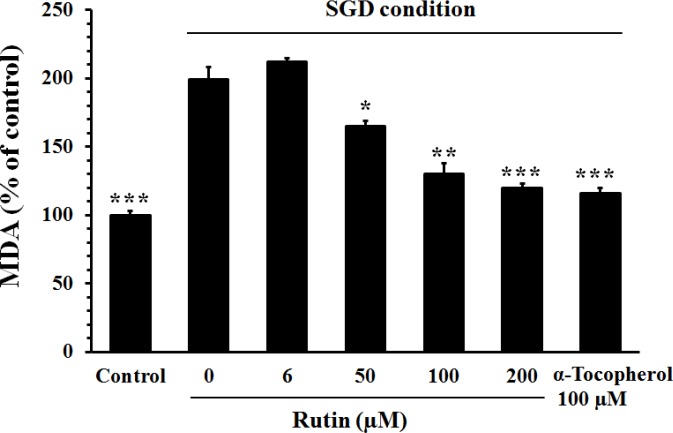
Effect of rutin on the lipid peroxidation in PC12 cells cultured in serum/glucose deprivation (SGD) condition. The cells were pretreated with rutin for 2 hr and then exposed for 12 hr to SGD condition. The lipid peroxidation was evaluated by measuring malondialdehyde (MDA). Pretreatment with rutin significantly decreased the SGD-induced lipid peroxidation. Data are mean±SEM (n=6). *P<0.05, **P<0.01, and ***P<0.001 compared to untreated cells (rutin 0 µM) cultured in SGD condition

Exposure of PC12 cells to SGD condition significantly increased the MDA level (199±9%, *P*<0.001) as compared to the cells incubated in normal medium (100±1.5%) (Figure 4). The level of MDA was significantly diminished in the cells pretreated with 100 µM of α-tocopherol (*P*<0.001) or rutin (50 μM, 100 μM, and 200 μM, *P*<0.05).

***Effect of rutin on DNA damage induced by SGD***

**Figure 5:**
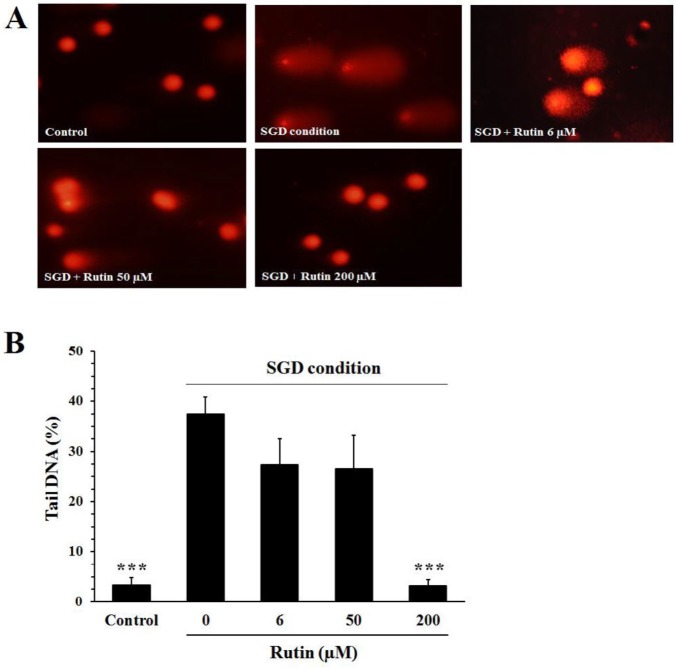
Effect of rutin on DNA damage in PC12 cells cultured in serum/glucose deprivation (SGD) condition. The cells were pretreated with rutin for 2 hr and then exposed for 12 hr to SGD condition. A: Representative micrographs of comets from PC12 cells. Undamaged cells (normal condition) have intact nucleus without a tail, while damaged cells in SGD condition show DNA fragmentation and have the appearance of a tail comet. B: The quantified data from the alkaline comet assay. A significant decrease in DNA fragmentation was observed in the cells pretreated with 200 µM of rutin. Data are mean±SEM of three independent experiments. ***P<0.001 compared to untreated cells (rutin 0 µM) cultured in SGD condition

Comet images from PC12 cells cultured in normal culture medium or SGD condition were shown in Figure 5A. Pc12 cells cultured in the normal medium (control) had intact nucleus without a tail, while the cells incubated in SGD condition displayed DNA fragmentation and had the appearance of a tail. DNA damage was quantified by determining the percent of DNA located in the comet tail. Exposure of the cells to SGD condition increased DNA fragmentation (37±3%) as compared to the cells incubated in normal medium (3.4±1.5%, *P*<0.001). A significant reduction in the SGD-induced DNA fragmentation was observed in the cells pretreated with 200 µM of rutin (3±1.2%, *P*<0.001).

***Effect of rutin on the level of proapoptotic and anti-apoptotic proteins***

**Figure 6:**
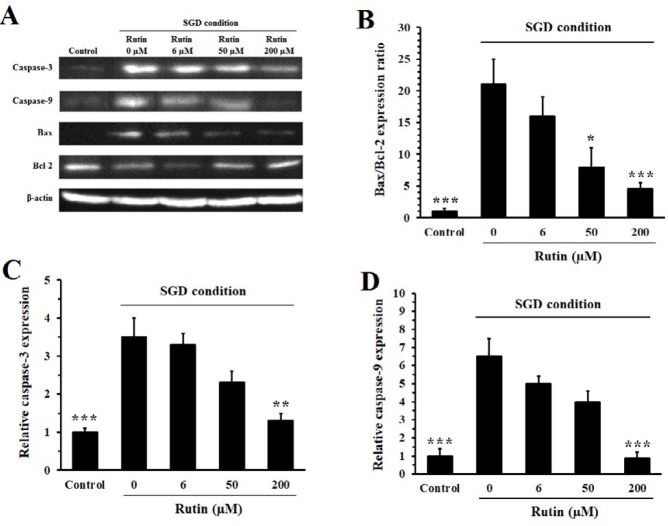
Effect of rutin on the expression of anti-apoptotic (Bcl-2) and proapoptotic (Bax, caspase-3, and caspase-9) proteins in PC12 cells cultured in serum/glucose deprivation (SGD) condition. The cells were pretreated with rutin for 2 hr and then exposed for 12 hr to SGD condition. Pretreatment with 200 μM of rutin significantly decreased the Bax/Bcl-2 ratio and the expression of caspase-3 and caspase-9 in GSD condition. Data are mean±SEM (n=3). *P<0.05, **P<0.01 and ***P<0.001 compared to untreated cells (rutin 0 µM) cultured in SGD condition

Culture of PC12 cells in SGD condition reduced the expression of Bcl-2 and enhanced the expression of Bax (Figure 6A). These changes led to a 21-fold increase in the ratio of Bax/Bcl-2 (Figure 6B), an indicator of mitochondria-mediated apoptosis. Pretreatment of ischemic cells with 50 and 200 μM of rutin decreased Bax/Bcl-2 ratio to 8±2 (*P*<0.05) and 4±1 (*P*<0.001), respectively. The expressions of cleaved forms of caspase-3 and caspase-9, as proapoptotic proteins, increased in the cells cultured in SGD condition. Rutin at 200 μM decreased the expression of caspase-3 (*P*<0.01) and caspase-9 (*P*<0.001), compared to untreated cells (Figure 6C and Figure 6D).

## Discussion

Current therapeutic options for brain ischemic attacks and other neurodegenerative disorders are limited and the search for new remedies has continued. The present work was aimed to evaluate the neuroprotective effect of rutin against SGD-induced DNA damage. The obtained results indicated that rutin is able to enhance the viability of neuronal cells in the nutrients deprivation condition through inhibiting oxidative stress, DNA damage, and apoptotic pathways.

Our observations that SGD condition enhanced the generation of ROS and lipid peroxidation were consistent with the well-known fact that oxidative stress plays a key role in the pathology of cerebral ischemia (2, 4). Pretreatment with rutin could decrease the level of intracellular ROS and inhibited lipid peroxidation. These effects of rutin at 200 µM was comparable to those of α-tocopherol (100 µM), an established antioxidant vitamin. Therefore, it seems that neuroprotective activity of rutin against SGD-induced cell death is mediated mainly through its antioxidant property. This is in agreement with other reports about antioxidant property of rutin in different experimental conditions (5, 6, 23, 24). For example, it has been reported that preventive action of rutin against isoproterenol-induced cardiac damage is mediated through its free radical scavenging effect and membrane stabilizing property (23). Also, Koda *et al. *(24) showed that beneficial effect of rutin on spatial memory in trimethyltin-induced neurotoxicity is attributed to its inhibitory effect on ROS production. 

Increased level of ROS is well-known to promote apoptosis by damaging proteins, lipids and nucleic acids (25). Apoptosis can be initiated by release of cytochrome C from mitochondria and subsequent activation of caspase-3 and caspase-9 (intrinsic pathway). Also, activation of cell death receptors can induce apoptosis through stimulation of caspase-8 (extrinsic pathway), which in turn activates caspase-3. Caspase-3 targets substrates that finally promote fragmentation of DNA. Mitochondria are considered as the main source of ROS involved in apoptosis following ischemia (25, 26). In response to apoptotic stimuli, the proapoptotic protein Bax moves from cytosol to the outer membrane of mitochondria, where it increases permeabilization of the membrane and promotes efflux of cytochrome C. The Bcl-2, as an anti-apoptotic protein, is located in the outer membrane of mitochondria and inhibits this effect of Bax. Therefore, the balance between these proteins (Bax/Bcl-2 ratio) is believed to be an important control point influencing the cellular fate (1, 25). In the present study, we observed that the ratio of Bax/Bcl-2 was increased in PC12 cells cultured in SGD condition. This effect was suppressed by pre-incubation of the cells with rutin. Also, rutin prevented the SGD-induced increase in the expression of caspase-3 and caspase-9 proteins. Similarly, Park *et al.* showed that rutin prevents the increase of Bax/Bcl-2 ratio in SH-SY5Y neuronal cells incubated with rotenone, a neurotoxin pesticide. Also, rutin protected the cells from rotenone-induced alteration of mitochondrial membrane potential and subsequent stimulation of caspase-3 and caspase-9 (27).

Results of comet assay revealed that rutin was able to protect PC12 cells against DNA fragmentation induced by SGD. In agreement with our findings, Undeger *et al.* demonstrated that rutin inhibited mitomycin C-induced DNA fragmentation in human lymphocytes (28). Also, it has been reported that rutin inhibits chromosomal and DNA damage induced by 2,5-hexanedione in rats (29).

## Conclusion

The present work showed that rutin reduces the generation of ROS and lipid peroxidation in neuronal cells cultured in nutrients deprivation condition. Also, it inhibits DNA fragmentation and apoptosis by decreasing the expressions of proapoptotic proteins (caspase-3, caspase-9, Bax) and increasing the level of Bcl-2, as an anti-apoptotic protein. Further studies may warrant the use of rutin as an appropriate neuroprotective agent for ischemic attacks and other neurodegenerative disorders.

## References

[1] Doyle KP, Simon RP, Stenzel-Poore MP (2008). Mechanisms of ischemic brain damage. Neuropharmacology.

[2] Li P, Stetler RA, Leak RK, Shi Y, Li Y, Yu W (2018). Oxidative stress and DNA damage after cerebral ischemia: Potential therapeutic targets to preserve the genome and improve stroke recovery. Neuropharmacology.

[3] Chauhan A, Chauhan V (2006). Oxidative stress in autism. Pathophysiology.

[4] Chamorro Á, Dirnagl U, Urra X, Planas AM (2016). Neuroprotection in acute stroke: targeting excitotoxicity, oxidative and nitrosative stress, and inflammation. Lancet Neurol.

[5] Na J-Y, Song K, Kim S, Kwon J (2016). Rutin protects rat articular chondrocytes against oxidative stress induced by hydrogen peroxide through SIRT1 activation. Biochem Biophys Res Commun.

[6] Singh S, Singh DK, Meena A, Dubey V, Masood N, Luqman S (2019). Rutin protects t-butyl hydroperoxide-induced oxidative impairment via modulating the Nrf2 and iNOS activity. Phytomedicine..

[7] Gao M, Ma Y, Liu D (2013). Rutin suppresses palmitic acids-triggered inflammation in macrophages and blocks high fat diet-induced obesity and fatty liver in mice. Pharm Res.

[8] Sadeghnia HR, Yousefsani BS, Rashidfar M, Boroushaki MT, Asadpour E, Ghorbani A (2013). Protective effect of rutin on hexachlorobutadiene-induced nephrotoxicity. Renal Failure.

[9] Ghorbani A (2017). Mechanisms of antidiabetic effects of flavonoid rutin. Biomed Pharmacother.

[10] Nassiri-Asl M, Shariati-Rad S, Zamansoltani F (2008). Anticonvulsive effects of intracerebroventricular administration of rutin in rats. Prog Neuro-Psychopharmacol Biol Psychiatry.

[11] Xu P-x, Wang S-w, Yu X-l, Su Y-j, Wang T, Zhou W-w (2014). Rutin improves spatial memory in Alzheimer’s disease transgenic mice by reducing Aβ oligomer level and attenuating oxidative stress and neuroinflammation. Behav Brain Res.

[12] Song H-l, Zhang X, Wang W-z, Liu R-h, Zhao K, Liu M-y (2018). Neuroprotective mechanisms of rutin for spinal cord injury through anti-oxidation and anti-inflammation and inhibition of p38 mitogen activated protein kinase pathway. Neural Regen Res.

[13] Hao G, Dong Y, Huo R, Wen K, Zhang Y, Liang G (2016). Rutin inhibits neuroinflammation and provides neuroprotection in an experimental rat model of subarachnoid hemorrhage, possibly through suppressing the RAGE–NF-κB inflammatory signaling pathway. Neurochem Res.

[14] Khan MM, Ahmad A, Ishrat T, Khuwaja G, Srivastawa P, Khan MB (2009). Rutin protects the neural damage induced by transient focal ischemia in rats. Brain Res.

[15] Pu F, Mishima K, Irie K, Motohashi K, Tanaka Y, Orito K (2007). Neuroprotective effects of quercetin and rutin on spatial memory impairment in an 8-arm radial maze task and neuronal death induced by repeated cerebral ischemia in rats. J Pharmacol Sci.

[16] Hillion JA, Takahashi K, Maric D, Ruetzler C, Barker JL, Hallenbeck JM (2005). Development of an ischemic tolerance model in a PC12 cell line. J Cereb Blood Flow Metab.

[17] Mousavi SH, Bakhtiari E, Hosseini A, Jamialahmadi K (2018). Protective effects of glucosamine and its acetylated derivative on serum/glucose deprivation-induced PC12 cells death: Role of reactive oxygen species. Res Pharm Sci.

[18] Jin Y, Tang X, Cao X, Yu L, Chen J, Zhao H (2018). 4-((5-(Tert-butyl)-3-chloro-2-hydroxybenzyl) amino)-2-hydroxybenzoic acid protects against oxygen-glucose deprivation/reperfusion injury. Life Sci.

[19] Lin W-C, Peng Y-F, Hou C-W (2015). Ferulic acid protects PC12 neurons against hypoxia by inhibiting the p-MAPKs and COX-2 pathways. Iran J Basic Med Sci.

[20] Asadpour E, Ghorbani A, Sadeghnia HR (2014). Water-soluble compounds of lettuce inhibit DNA damage and lipid peroxidation induced by glucose/serum deprivation in N2a cells. Acta Pol Pharm.

[21] Hadjzadeh M, Tavakol Afshari J, Ghorbani A, Shakeri M (2006). The effects of aqueous extract of garlic (Allium sativum L) on laryngeal cancer cells (Hep-2) and L929 cells in vitro. J Med Plants.

[22] Sadeghnia HR, Kamkar M, Assadpour E, Boroushaki MT, Ghorbani A (2013). Protective effect of safranal, a constituent of Crocus sativus, on quinolinic acid-induced oxidative damage in rat hippocampus. Iran J Basic Med Sci.

[23] Prince PSM, Priya S (2010). Preventive effects of rutin on lysosomal enzymes in isoproterenol induced cardio toxic rats: Biochemical, histological and in vitro evidences. Eur J Pharmacol.

[24] Koda T, Kuroda Y, Imai H (2008). Protective effect of rutin against spatial memory impairment induced by trimethyltin in rats. Nutr Res.

[25] Broughton BR, Reutens DC, Sobey CG (2009). Apoptotic mechanisms after cerebral ischemia. Stroke.

[26] Bhat AH, Dar KB, Anees S, Zargar MA, Masood A, Sofi MA (2015). Oxidative stress, mitochondrial dysfunction and neurodegenerative diseases; a mechanistic insight. Biomed Pharmacother.

[27] Park S-E, Sapkota K, Choi J-H, Kim M-K, Kim YH, Kim KM (2014). Rutin from Dendropanax morbifera Leveille protects human dopaminergic cells against rotenone induced cell injury through inhibiting JNK and p38 MAPK signaling. Neurochem Res.

[28] Ündeğer Ü, Aydın S, Başaran AA, Başaran N (2004). The modulating effects of quercetin and rutin on the mitomycin C induced DNA damage. Toxicol Lett.

[29] Muhammad A, Arthur DE, Babangida S, Erukainure OL, Malami I, Sani H (2018). Modulatory role of rutin on 2,5-hexanedione-induced chromosomal and DNA damage in rats: validation of computational predictions. Drug Chem Toxicol.

